# Comparison of Different Lymph Node Staging Systems in Patients With Resectable Colorectal Cancer

**DOI:** 10.3389/fonc.2018.00671

**Published:** 2019-01-15

**Authors:** Jun-Peng Pei, Chun-Dong Zhang, Yu-Chen Fan, Dong-Qiu Dai

**Affiliations:** ^1^Department of Gastrointestinal Surgery, The Fourth Affiliated Hospital of China Medical University, Shenyang, China; ^2^Department of Gastrointestinal Surgery, Graduate School of Medicine, University of Tokyo, Tokyo, Japan; ^3^Cancer Center, The Fourth Affiliated Hospital of China Medical University, Shenyang, China

**Keywords:** log odds, lymph node ratio, N staging, colorectal cancer, survival analysis

## Abstract

**Background and Objectives:** Currently, the United States Joint Commission on Cancer (AJCC) N staging, lymph node positive rate (LNR), and log odds of positive lymph nodes (LODDS) are the main lymph node (LN) staging systems. However, the type of LN staging system that is more accurate in terms of prognostic performance remains controversial. We compared the prognostic accuracy of the three staging systems in patients with CRC and determine the best choice for clinical applications.

**Methods:** From the Surveillance, Epidemiology, and End Results (SEER) database, 56,747 patients were identified who were diagnosed with CRC between 2004 and 2013. Akaike's Information Criterion (AIC) and Harrell's Consistency Index (c-index) were used to assess the relative discriminative abilities of different LN staging systems.

**Results:** In 56,747 patients, when using classification cut-off values for evaluation, the LNR of Rosenberg et al. showed significantly better predictive power, especially when the number of dissected lymph nodes (NDLN) were insufficient. When analyzed as a continuous variable, the LODDS staging system performed the best and was not affected by the NDLN.

**Conclusions:** We suggest that the LNR of Rosenberg et al. should be introduced into the AJCC system as a supplement when the NDLN is insufficient until the optimal LODDS cut-off values are calculated.

## Introduction

Colorectal cancer (CRC) is the third most commonly diagnosed cancer in men and women in the United States (1). Lymph node (LN) metastasis is an important prognostic factor associated with overall survival (OS) (2). Therefore, in order to accurately describe LN status, a variety of LN staging systems have been proposed. The most representative of these LN staging systems are the American Joint Committee on Cancer/Union for International Cancer Control (AJCC/UICC) eighth edition N staging (3), lymph node ratio (LNR) and the log odds of positive lymph nodes (LODDS).

The goal of cancer staging systems is to group patients with similar prognosis. Rice et al. defined the characteristics of a good staging system as: (a) the patient survival rate decreases as the stage group increases (Monotonicity), (b) the groups have clearly different survival rates (Distinctiveness), and (c) within a group, the survival rate is similar (Homogeneity) (4). Currently, the most widely accepted LN staging system is the AJCC/UICC 8th N staging, which is based on the absolute number of positive lymph nodes (NPLN). Its classification system is: pN0: no LN metastasis; pN1a: 1 metastatic LN; pN1b: 2–3 metastatic LNs; pN2a: 4–6 metastatic LNs; pN2b: ≥7 metastatic LNs (3).

Many studies have shown that OS is closely related to the NDLN in resectable surgery in patients with CRC, and a greater NDLN could provide more accurate staging and longer survival (5–7). The AJCC/UICC 8th N staging system recommends that at least 12 LNs in tumor specimens must be resectable and histopathologically evaluated to fully assess LN status. However, despite the availability of accurate recommendations, the recommended cut-off values for the NDLN needed varies widely among published studies, with the median ranging between 6 and 13, which results in staging migration and can affect further treatment for CRC (8, 9). In addition to surgeons, pathologists have also played a significant role in determining the status of LN in resected specimens (10). Therefore, in order to reduce staging migration, two new LN staging systems have been proposed.

LNR is defined as the ratio of NPLN relative to the NDLN. Recently, some scholars have reported that LNR has been shown to have a strong independent prognostic value in rectal and colon cancer (11, 12). These results were also shown in patients with lung, breast, and gastric cancer (13–15). Berger et al. first proposed that LNR has a higher prognostic impact in patients with colon cancer. They believed that LNR could reduce staging migration in patients with an insufficient NDLN (16). Rosenberg et al. also suggested that LNR should include routine histopathology reports because of their higher prognostic impact on colon cancer than AJCC/UICC N staging (17). However, some experts believe that when the NDLN is not sufficient, LNR cannot completely eliminate staging migration (18, 19). In addition, when LNR is an extreme value (LNR = 0 or 1), it does not accurately predict prognosis (12).

LODDS is another innovative N staging system. LODDS is defined as the logarithm of the ratio between the probability of being a positive LN and the probability of being a negative LN when an LN is retrieved (5, 20, 21). The formula for the LODDS system is log{(NPLN + 0.5)/(NDLN - NPLN + 0.5)}. “0.5” appears twice in the formula to avoid dividing by 0 and avoid having many patients with a LODDS of 0. According to previous reports, the use of LODDS has reduced the risk of staging migration in gastric, breast, colon, and pancreatic cancer in recent years (22–25). After comparing the prognostic utility of the LODDS system with the LNR system and AJCC/UICC N staging in patients with CRC, Persiani et al. showed that the LODDS system performed better (24). Wang et al. used the Surveillance, Epidemiology, and End Results (SEER) data to study the LODDS system in stage III colon cancer cases and concluded that LODDS also performed better than LNR and AJCC/UICC N staging in predicting prognosis (26).

The aim of this study was to compare the ability of different LN staging systems to predict OS in patients with resectable CRC to identify the most accurate system for application in clinical practice.

## Materials and Methods

### Patients

In this retrospective analysis, we used data from the SEER linked database. The SEER Program of the National Cancer Institute is an authoritative source of information on cancer incidence and survival in the United States (U.S.) that is updated annually. SEER currently collects and publishes cancer incidence and survival data from population-based cancer registries covering approximately 34.6 percent of the U.S. population. Data from SEER was used to identify patients with CRC diagnosed between 2004 and 2013. Among the 90,529 patients diagnosed with CRC between these years, patients with the following characteristics were included: (a) the patients were over 18 years old; (b) CRC was the first and only malignant tumor; (c) surgical resection was performed; (d) there was complete staging information; and (e) no neoadjuvant chemoradiation was used in treatment. The final study sample contained 56,747 patients.

### LN Staging Systems

We analyzed LNR and LODDS as both continuous and categorical variables. When used as categorical variables, different researchers have developed different optimal cut-off values. For the LNR staging system, we used cut-off values from Berger et al. and Rosenberg et al. Berger et al. considered 0.05, 0.19, and 0.39 as the best cut-off values, and divided the LNR into four groups as follows: LNR1 < 0.05; 0.05 ≤ LNR2 < 0.19; 0.19 ≤ LNR3 < 0.39; and 0.39 ≤ LNR4 ≤ 1.00 (16). Rosenberg et al. calculated the best cut-off values between groups as 0.17, 0.41 and 0.69, and divided the LNR into five subgroups as follows: LNR0 = 0.00; 0.01 ≤ LNR1 ≤ 0.17; 0.18 ≤ LNR2 ≤ 0.41; 0.42 ≤ LNR3 ≤ 0.69; and LNR4 ≥ 0.70 (17). For the LODDS staging system, we used the ideal cut-off values from Persiani et al. and Wang et al. Persiani et al. divided LODDS into three groups as follows: LODDS1 ≤ −1.36;−1.36 < LODDS2 ≤ −0.53; LODDS3 >−0.53 (24). Wang et al. divided LODDS into five groups as follows: LODDS1 < −2.2;−2.2 ≤ LODDS2 < −1.1;−1.1 ≤ LODDS3 < 0.0; 0.0 ≤ LODDS4 < 1.1; LODDS5 ≥ 1.1 (26) (Table 1).

**Table 1:** Basic characteristics of different lymph node staging systems.

**AJCC 8th N stage (3)**	**LNR, Berger et al. (16)**	**LNR, Rosenberg et al. (17)**	**LODDS, Wang et al. (26)**	**LODDS, Persiani et al. (24)**
pN0	LNR1 < 0.05	LNR0 = 0.00	LODDS1 < −2.2	LODDS1 ≤ −1.36
pN1a	0.05 ≤ LNR2 < 0.19	0.01 ≤ LNR1 ≤ 0.17	−2.2 ≤ LODDS2 < −1.1	−1.36 < LODDS2 ≤ −0.53
pN1b	0.19 ≤ LNR3 < 0.39	0.18 ≤ LNR2 ≤ 0.41	−1.1 ≤ LODDS3 < 0.0	LODDS3 > −0.53
pN2a	0.39 ≤ LNR4 ≤ 1.00	0.42 ≤ LNR3 ≤ 0.69	0.0 ≤ LODDS4 < 1.1
pN2b		LNR4 ≥ 0.70	LODDS5 ≥ 1.1

*AJCC, American Joint Committee on Cancer; LNR, lymph node ratio; LODDS, log odds of positive lymph nodes*.

### Statistical Analysis

We used the Kaplan-Meier method to estimate OS and tested it using the log-rank procedure. Odds ratio (OR) and 95% confidence intervals (95% CI) are presented. We used the Akaike Information Criterion (AIC) and the Harrell Consistency Index (c-index) to assess the relative discriminative power of different LN staging systems. A value of c = 0.5 indicates no predictive power, and a value of c = 1 indicates complete differentiation. In general, a predictive model with a low AIC indicates a better model fit, while a high c-index indicates a better discriminating ability. All analyses were carried out with SPSS version 22.0 and R version 3.50. For all analysis, *P* < 0.05 was considered significant, and all tests were two-tailed.

## Results

### Patient Characteristics

Table 2 shows clinical and histopathological characteristics for the study population. The cohort consisted of 27,507 males (48.5%) and 29,240 females (51.5%). The median age ± standard deviation was 66.0 ± 13.3 years. There were 22,723 (40.5%) patients with CRC who had LN metastases and 34,024 (59.5%) patients with no LN metastases. The mean ± standard deviation of NDLN and NPLN in the whole cohort were 16.9 ± 9.8 and 1.6 ± 3.3, respectively. 10,613 (18.7%) subjects had tumor located in the rectum and 46,134 (81.3%) were in the colon. In the univariate analysis, the age of diagnosis, histological grade, pT stage, tumor size, and NDLN were significantly correlated with prognosis.

**Table 2:** Clinical and histopathological characteristics for the entire population.

**Variables**	**N (%)**	**Univariate analysis**	
		**5-year OS (%)**	***P-*value**
Age, years			< 0.001
≤ 65	26,305 (46.4)	85.3
> 65	30,442 (53.6)	72.6
Gender			0.353
Male	27,507 (48.5)	78.5
Female	29,240 (51.5)	78.7
Tumor location			0.763
Rectum	10,613 (18.7)	78.3
Colon	46,134 (81.3)	78.5
Histologic grade			< 0.001
Well differentiated	5,382 (9.5)	87.6
Moderately differentiated	41,004 (72.3)	80.2
Poorly differentiated	9,609 (16.9)	66.9
Undifferentiated	752 (1.3)	65.4
Tumor size, cm			< 0.001
≤ 5	35,672 (62.9)	80.6
> 5	16,259 (28.7)	72.2
Unknown	4,816 (8.5)	85.6
AJCC 8th T stage			< 0.001
pT1	8,022 (14.1)	95.5
pT2	9,957 (17.5)	91.6
pT3	32,726 (57.7)	75.7
pT4	6,042 (10.7)	51.6
NDLN			< 0.001
Inadequate (*n* < 12)	16,699 (29.4)	76.2
Adequate (*n* ≥ 12)	40,048 (70.6)	79.7

*N, number; OS, overall survival rate; NDLN, the number of dissected lymph nodes; AJCC, American Joint Committee on Cancer*.

### Survival

**Figure 1:**
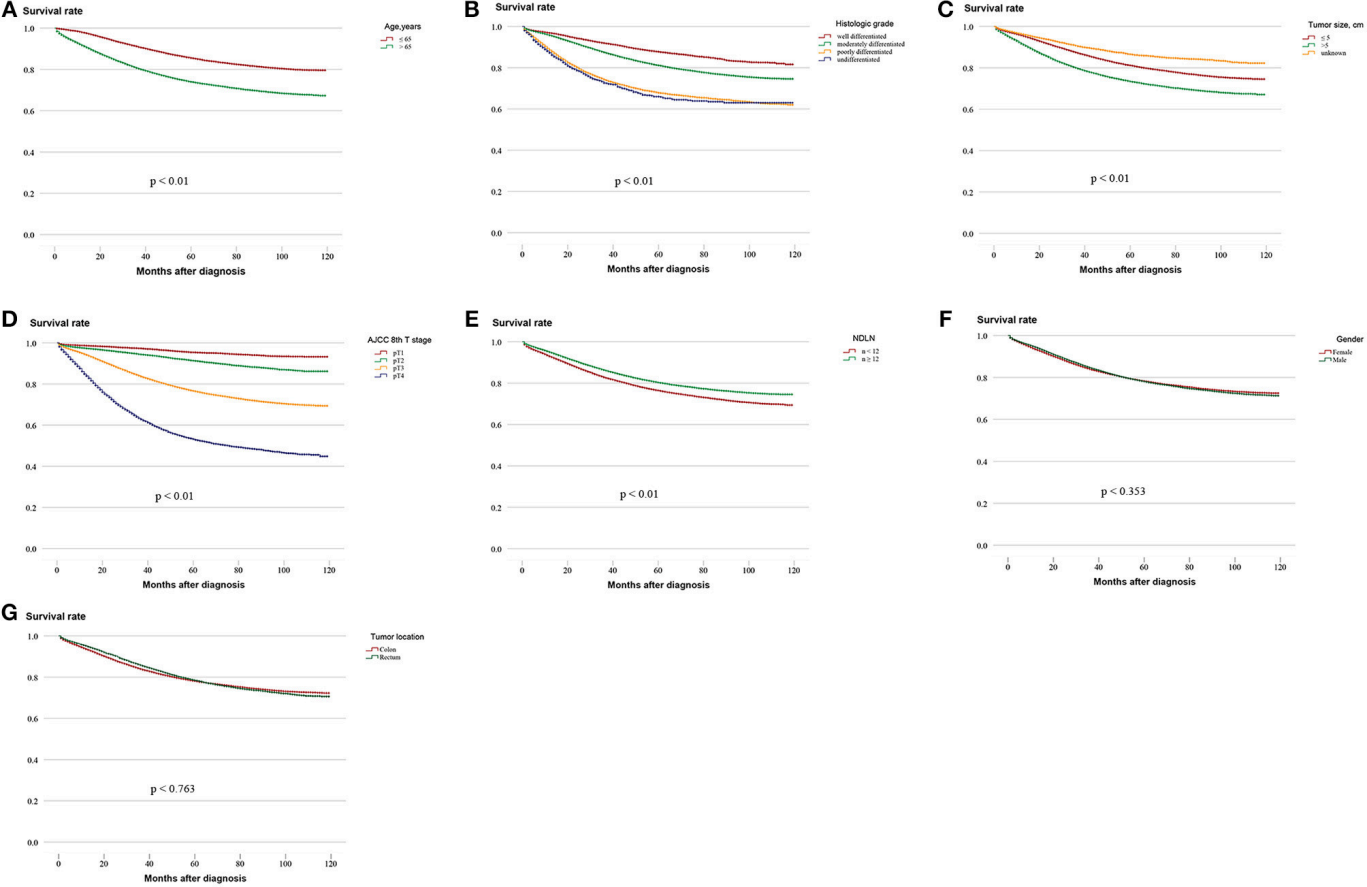
Kaplan–Meier survival curves for five-year OS stratified by different prognostic factors with statistical significance based on the **(A)** Age, **(B)** Histologic grade, **(C)** Tumor size, **(D)** AJCC 8th T stage, **(E)** NDLN, **(F)** Gender, and **(G)** Tumor location. (AJCC, American Joint Committee on Cancer; NDLN, the number of dissected lymph nodes).

**Figure 2:**
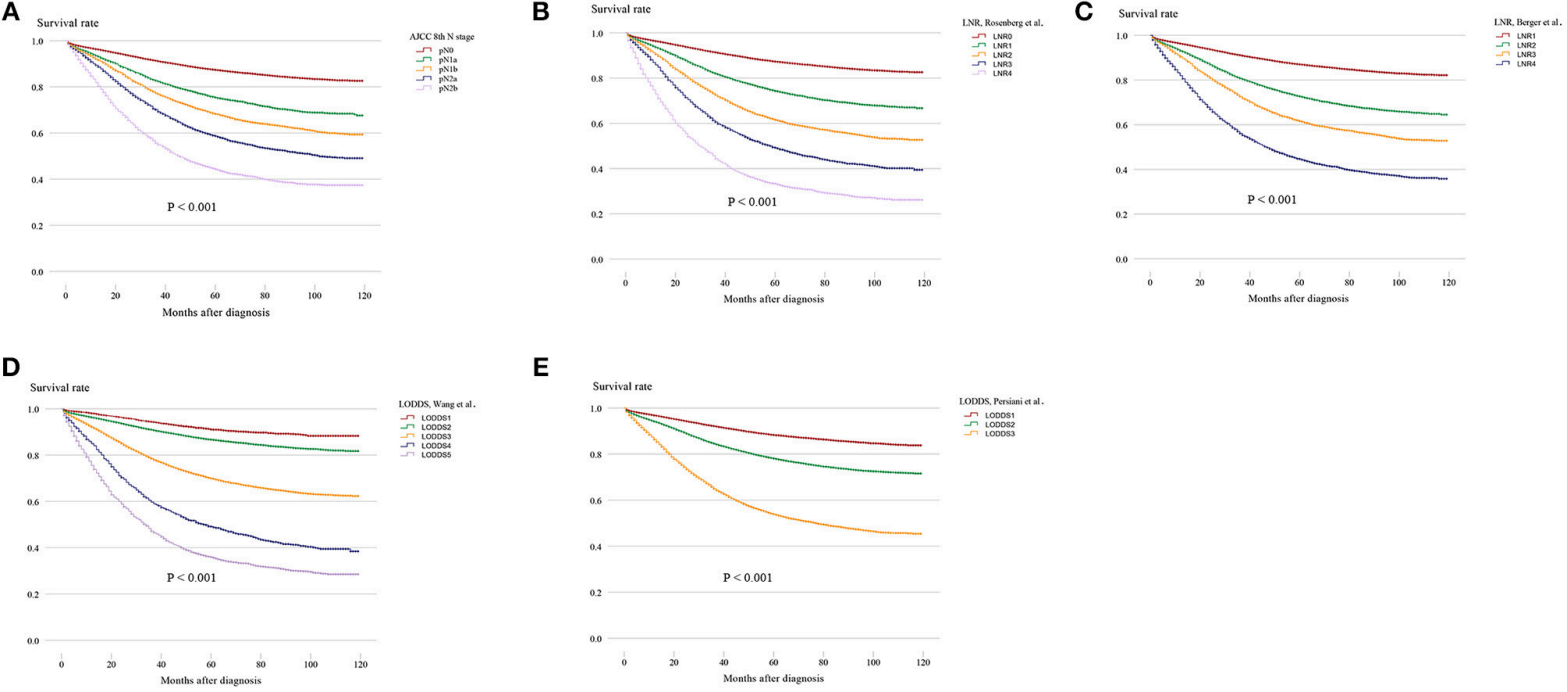
Kaplan–Meier survival curves for five-year OS stratified by LN categories based on the **(A)** AJCC 8th N stage, **(B)** LNR of Rosenberg et al. **(C)** LNR of Berger et al. **(D)** LODDS of Wang et al. and **(E)** LODDS of Persiani et al. (LN, lymph node; AJCC, American Joint Committee on Cancer; LNR, lymph node ratio; LODDS, log odds of positive lymph nodes).

Survival analysis was performed on the factors in the univariate analysis (Figures 1A–G). The 5-year OS of patients with an adequate NDLN was 79.7% and with an inadequate NDLN was 76.2% (*P* < 0.001; Figure 1E). The 5-year OS of patients with tumor located in the rectum was 78.3% and in the colon was 78.5%. The tumor location was not significant in predicting prognosis (*P* = 0.763; Figure 1G). Therefore, we grouped rectal and colon cancer together. The 5-year OS of different histological grades were 87.6% for well differentiated, 80.2% for moderately differentiated, 66.9% for poorly differentiated, and 65.4% for undifferentiated (*P* < 0.001; Figure 1B). No significant difference was found between poorly differentiated and undifferentiated tumors (*P* = 0.148). Kaplan-Meier survival curves and survival data based on different LN staging systems are shown in Figure 2 and Table 3 for all patients. The AJCC/UICC N staging system divided patients into five different prognostic groups and the 5-year OS for each subgroup were: pN0 = 87.2%, pN1a = 75.2%, pN1b = 68.1%, pN2a = 58.3%, and pN2b = 44.1% (*P* < 0.001; Figure 2A). The 5-year OS of the LNR subgroups according to the Rosenberg et al. criteria were LNR0 = 87.2%, LNR1 = 74.1%, LNR2 = 61.3%, LNR3 = 48.9%, and LNR4 = 33.0% (*P* < 0.001; Figure 2B), and the 5-year OS according to the Berger et al. criteria were LNR1 = 86.9%, LNR2 = 72.4%, LNR3 = 61.3%, and LNR4 = 44.3% (*P* < 0.001; Figure 2C). Finally, the 5-year OS of LODDS based on the classification by Wang et al. were LODDS1 = 91%, LODDS2 = 86.5%, LODDS3 = 69.7%, LODDS4 = 48.8%, and LODDS5 = 35.6% (*P* < 0.001; Figure 2D) and those using the criteria by Persiani et al. were LODDS1 = 88.2%, LODDS2 = 77.9%, LODDS3 = 53.6% (*P* < 0.001; Figure 2E). Significant survival differences were detected between the subgroups of each staging system (Figure 2,Table 3).

**Table 3:** Five-year overall survival and 95% confidence interval according to different LN staging.

**Staging systems**	**N (%)**	**OR (95 % CI)**	**5-year OS (%)**
**AJCC 8th N stage (3)**			
pN0	34,024 (60.0)	1.00 (Reference)	87.2
pN1a	6,975 (12.3)	2.06 (1.95–2.17)	75.2
pN1b	7,149 (12.6)	2.76 (2.63–2.90)	68.1
pN2a	4,764 (8.4)	3.82 (3.63–4.02)	58.3
pN2b	3,835 (6.8)	5.80 (5.52–6.10)	44.1
**LNR, Berger et al. (16)**			
LNR1	36,041 (63.5)	1.00 (Reference)	86.9
LNR2	10,058 (17.7)	2.26 (2.16–2.36)	72.4
LNR3	5,594 (9.9)	3.34 (3.19–3.51)	61.3
LNR4	5,054 (8.9)	5.62 (5.38–5.88)	44.3
**LNR, Rosenberg et al. (17)**			
LNR0	34,024 (60.0)	1.00 (Reference)	87.2
LNR1	11,520 (20.3)	2.41 (2.05–2.24)	74.1
LNR2	6,659 (11.7)	3.44 (3.29–3.61)	61.3
LNR3	2,919 (5.1)	5.06 (4.78–5.35)	48.9
LNR4	1,625 (2.9)	7.99 (7.49–8.53)	33.0
**LODDS, Wang et al. (26)**			
LODDS1	3,707 (6.5)	1.00 (Reference)	91.0
LODDS2	29,557 (52.1)	1.57 (1.41–1.75)	86.5
LODDS3	19,761 (34.8)	3.84 (3.45–4.27)	69.7
LODDS4	1,578 (2.8)	7.70 (6.80–8.70)	48.8
LODDS5	2,144 (3.8)	11.00 (9.80–12.35)	35.6
**LODDS, Persiani et al. (24)**			
LODDS1	24,983 (44.0)	1.00 (Reference)	88.2
LODDS2	21,423 (37.8)	1.97 (1.88–2.06)	77.9
LODDS3	10,341 (18.2)	4.81 (4.60–5.02)	53.6

*N, number; OR, Odds Ratio; 95% CI, 95% confidence interval; LNR, lymph node ratio; LODDS, log odds of positive lymph nodes; AJCC, American Joint Committee on Cancer*.

### Prognostic Accuracy of Different LN Staging Systems

The AIC and c-index were used to estimate the prognostic discriminative ability of different LN staging systems (Table 4). First, the LN status was evaluated as a categorical variable to analyze the prognostic discriminating power of different LN staging systems. In the whole population, two LNR staging systems showed better prognostic performance than other staging systems, with the LNR from Rosenberg et al. (c-index: 0.669, AIC: 287984.1) showing the best prognostic performance. The LNR of Berger et al. (c-index: 0.666, AIC: 288125.3) and AJCC/UICC N staging (c-index: 0.666; AIC: 288397.0) had similar prognostic performances. In addition, the two LODDS (Wang et al.: c-index: 0.659, AIC: 288619.9; Persiani et al.: c-index: 0.659, AIC: 288994.6) staging systems performed relatively poorly. Further analysis based on different NDLN showed that when the NDLN was insufficient (NDLN < 12), the LNR of Rosenberg et al. (c-index: 0.649, AIC: 85842.9) still maintained the best prognostic performance. However, when the NDLN is sufficient (NDLN ≥ 12), AJCC/UICC N staging (c-index: 0.647; AIC: 85899.4) is the best prognostic model. In contrast, both LODDS staging systems showed the worst prognosis performance regardless of the adequacy of the NDLN.

**Table 4:** Prognostic performance of different lymph node staging systems before and after stratifying for NDLN.

**NDLN**					
**Variables**	**ALL (*n* = 56, 747)**		**≥ 12 (*n* = 16, 699)**		**< 12 (*n* = 40, 048)**	
	**C Index (95% CI)**	**AIC**	**C Index (95% CI)**	**AIC**	**C Index (95% CI)**	**AIC**
PLN (continuous)	0.668 (0.663–0.672)	290576.3	0.682 (0.677–0.688)	186085.5	0.648 (0.641–0.655)	86189.8
LNR (continuous)	0.673 (0.668–0.677)	288763.7	0.684 (0.679–0.690)	185052.7	0.651 (0.644–0.658)	86050.7
LODDS (continuous)	0.682 (0.677–0.687)	287860.5	0.691 (0.685–0.697)	184338.2	0.652 (0.644–0.661)	85970.4
AJCC 8th N stage (3)	0.666 (0.662–0.671)	288397.0	0.681 (0.675–0.686)	184632.6	0.647 (0.640–0.654)	85899.4
LNR, Rosenberg et al. (17)	0.669 (0.664–0.673)	287984.1	0.679 (0.673–0.684)	184496.2	0.649 (0.642–0.656)	85842.9
LNR, Berger et al. (16)	0.666 (0.662–0.670)	288125.3	0.674 (0.669–0.679)	184686.9	0.639 (0.632–0.646)	85856.0
LODDS, Wang et al. (26)	0.659 (0.655–0.664)	288619.9	0.665 (0.660–0.670)	184888.2	0.629 (0.621–0.636)	86265.7
LODDS, Persiani et al. (24)	0.659 (0.654–0.663)	288994.6	0.673 (0.668–0.678)	184899.7	0.616 (0.609–0.623)	86388.1

*NDLN, The number of dissected lymph nodes; AIC, Akaike's Information Criterion; C Index, Harrell's consistency Index; LNR, lymph node ratio; LODDS, log odds of positive lymph nodes; AJCC, American Joint Committee on Cancer; PLN, positive lymph node*.

To assess whether the ability of the predicted prognosis of different LN staging systems was affected by artificially determined cut-off values, the LN status was modeled as a continuous variable for repeated analysis. The results showed that the LODDS system was superior to other staging systems and was not affected by the NDLN. It is worth noting that PLN always showed the worst prognostic discriminative ability regardless of whether the NDLN was sufficient.

**Figure 3:**
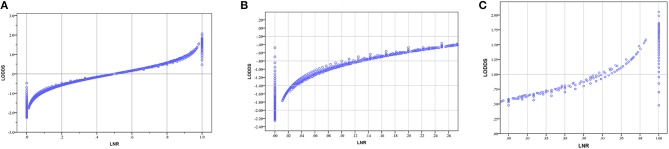
**(A)** The scatter plots of LODDS vs. LNR; **(B)** The magnified view of **(A)** for LNR between 0 to 0.25; **(C)** The magnified view of A for LNR between 0.75 to 1. (LNR, lymph node ratio; LODDS, log odds of positive lymph nodes).

We created scatter plots to explain the relationship between LNR and LODDS. As shown in Figure 3A, when patients have different LNR, the LODDS has a one-to-one mapping value for each LNR, and as the LNR increases, the value of LODDS increases. This indicates a close correlation between LODDS and LNR (except when LNR = 0 or 1). Thus, both contain the same prognostic information. However, as shown in Figures 3B,C, when the LNR is close to 0 or 1, the value of LODDS is heterogeneous.

## Discussion

Regional LN metastasis of malignant tumors is one of the main metastatic patterns of CRC. LN status is also considered to be one of the most important prognostic parameters for recurrence and death after CRC resection. Therefore, accurate staging of LN status can more accurately predict cancer risk and lead to the development of postoperative treatment options for patients with CRC (16). A number of LN staging systems have been proposed to accurately describe LN status, including AJCC/UICC N staging, LNR, and the LODDS staging systems. Among them, the AJCC/UICC N staging system is widely recognized and used in clinical practice, but some scholars question its accuracy (19, 27–31). Some researchers have shown that the NPLN is significantly correlated with the NDLN, especially when the NDLN is insufficient, which may lead to the missed PLN, resulting in staging migration (6, 7, 16). LNR is a ratio-based LN status estimation method that considers both the NPLN and NPDLN. Many researchers have demonstrated that it is a better independent prognostic factor than the AJCC/UICC N staging in rectal cancer or colon cancer (27–31). Ozawa et al. studied the prognostic ability of LNR in stage IV CRC and found that patients with the same AJCC/UICC N staging group had 23% higher OS in the low LNR group than the high LNR group (32). This further illustrates that subgroups of patients with the same AJCC/UICC N stage can be divided into significantly different prognostic subgroups by the LNR system, and other studies have reached similar conclusions (17, 18). LODDS is another staging system that describes the LN status and has great potential to further improve the accuracy of LN staging for predicting prognosis. Persiani et al. used multivariate regression analysis to compare the accuracy of different LN staging systems in estimating the prognosis of colon cancer (24). That study demonstrated that LODDS is an independent prognostic factor, further showing that LODDS is more accurate than LNR in assessing colon cancer survival, and other researchers have used similar methods to draw similar conclusions (5, 21, 26, 33). However, they did not use statistical methods to directly compare the discriminative ability of different LN staging system models.

In our study, we used two statistical indicators, the AIC and the c-index, to analyze the relative discriminative ability of different LN staging systems in predicting CRC survival in a CRC patient population. We first analyzed LN status as a continuous variable. We found that LODDS is superior to PLN and LNR. When we analyzed LN status as a categorical variable, we showed that the two LNR staging systems were superior to other staging systems.

There is still controversy regarding the categorical cut-off values for different LN staging systems. The reason for heterogeneity in the cut-off values is multifactorial. First, different studies used different statistical methods to determine these optimal cut-off values. For example, Song et al. used log-rank statistical methods (34), Rosenberg et al. used categorical and regression tree techniques (17), Berger et al. used the quartile method (16), Kornprat et al. used the receiver operating characteristic (ROC) statistical method (35), and Wang et al. used the X-tile program (26). In addition, different countries and research institutions, differences in patient numbers, and different average NDLN also lead to the diversity in cut-off values.

In addition to LN status and categorical cut-off values, many studies have shown that the NDLN has a significant impact on patient prognosis. Le Voyer et al. showed that an increase in the NDLN was significantly associated with improved OS (7). The National Comprehensive Cancer Network (NCCN) guidelines recommend at least 12 NDLN for accurate staging. However, the NDLN in clinically resected specimens can vary greatly. In our study, the proportion of patients with insufficient NDLN reached 29.4%. In view of this, we conducted a subgroup study based on different NDLN to analyze the prognostic accuracy of each LN staging system. We divided patients into two subgroups according to the NDLN: NDLN < 12 and NDLN ≥ 12.

Therefore, we conducted a comprehensive study based on LN status (continuous variable and categorical variable) and the NDLN. When analyzed as a categorical variable, the LNR of Rosenberg et al. (17) was the best staging system when the NDLN < 12. However, in patients with NDLN ≥ 12, AJCC/UICC N staging is the most accurate system for predicting patient outcomes. When analyzed as a continuous variable, LODDS showed the best discrimination ability regardless of the NDLN.

Many studies have shown that evaluating the LN status as a continuous variable reveals its true performance, so LODDS is a more accurate staging system than LNR in predicting CRC patient OS (36). We further illustrated the relationship between LNR and LODDS through scatter plots. Figure 3 shows that the overall trend of LNR and LODDS is consistent. However, when the LNR is around 0 or 1, the value of LODDS is heterogeneous, indicating that LODDS has a better discriminating power for patients with very low or high LNR. Some researchers believe that because of the lack of consensus on the cut-off values of different LN staging systems, LN status should be treated as a continuous variable (36). However, we believe that ignoring the cut-off values and using the LN status as a continuous variable cannot be applied in clinical practice. Thus, it has only theoretical value and no practical clinical value. Although LODDS is the best staging system, LODDS has no advantage over other staging systems when considering the impact of categorical cut-off values on staging systems. Therefore, optimal cut-off values should be calculated to make the LODDS staging system more useful for clinical practice.

The innovations of this study are as follows. First, the SEER data offers the unique opportunity to study prognostic elements in a larger number of patients. Second, in seeking the best staging system, we took the cut-off values of each staging system into account. However, there are limitations to our results, and we advise appropriate caution in their interpretation. This is a retrospective study based on the SEER database, so there will inevitably be some selection bias. The SEER database lacks some clinical information such as operative time, specific surgical procedures, lymph and/or vascular invasion, and specific locations of LN metastasis. Additionally, these results may not be applicable to other populations as they were based on Western patient data. Whether the use of this staging system could be applied to daily practice in Eastern countries, therefore, requires to be further validated. However, these shortcomings are common to any retrospective and population-based research. Finally, we believe that the patient data for this study is large and these shortcomings can be largely compensated by long-term follow-up.

## Conclusions

In conclusion, we believe that regardless of the adequacy of the NDLN, LODDS is the most accurate staging system for predicting the survival of patients with CRC. However, the best LODDS cut-off values that can be applied to clinical practice have not been calculated. Therefore, the LNR staging system of Rosenberg et al. with cut-off values of 0.17, 0.41, and 0.69 should be introduced to the AJCC/UICC system as supplements when the NDLN are insufficient.

## Author Contributions

J-PP, C-DZ, and D-QD designed this study. J-PP and C-DZ performed search and collected data. Y-CF re-checked data. J-PP and C-DZ performed analysis. J-PP wrote the manuscript. C-DZ and D-QD reviewed the manuscript.

### Conflict of Interest Statement

The authors declare that the research was conducted in the absence of any commercial or financial relationships that could be construed as a potential conflict of interest.
